# The Human Oocyte Preservation Experience (HOPE) a phase IV, prospective, multicenter, observational oocyte cryopreservation registry

**DOI:** 10.1186/1477-7827-7-53

**Published:** 2009-05-27

**Authors:** Diego Ezcurra, Jennifer Rangnow, Maryellen Craig, Joan Schertz

**Affiliations:** 1 EMD Serono Inc, Rockland, Massachusetts, USA

## Abstract

**Background:**

It has been recommended by the American Society of Clinical Oncology and the American Society of Reproductive Medicine that options to preserve fertility be presented at the outset of treatment for cancer. This recommendation may have arisen, in part, to the increasing survival of patients with cancer and the realization that certain forms of cancer treatment can lead to infertility. One option for these patients, particularly those with ethical or religious objections to freezing embryos is oocyte cryopreservation. However universal acceptance of these procedures has yet to be established, most likely due to a poor history of success and concerns that there has yet to be a comprehensive approach to evaluating these techniques. In light of this, a registry of patients undergoing oocyte cryopreservation, called the HOPE registry, is being implemented.

**Discussion:**

The intent of the HOPE Registry is to enroll approximately 400 women of reproductive age who will undergo thawing/warming of oocytes and subsequent transfer. Data from the patients enrolled will be collected via a uniform, standardized form and will document important parameters such as demographics, laboratory procedures and outcomes, including following the outcomes of babies born for one year after birth. The results of the registry will be published on a yearly basis.

**Summary:**

A patient registry has been established in order to systematically document the techniques and outcomes of oocyte cryopreservation procedures. The results will be published in order to provide a widely accessible resource that will allow patients who are considering these procedures validated information in order to make informed decisions as to how their treatment will proceed.

## Background

The American Cancer Society recently reported a 32% increase in cancer survival rates for all cancers diagnosed from 1996 and 2003 [1]. In the past five years, the number of novel cancer treatments has increased over 50%, and it is estimated that >1,400 new drug applications for cancer treatment will be submitted in 2008 [2]. With heightened public awareness, more aggressive screening and treatment innovation, survival rates are anticipated to increase steadily, allowing more patients to look forward to a near-normal life after cancer.

Unfortunately, cancer treatment often results in infertility [3]. For example, Goodwin et al. estimated that 53 to 89% of breast cancer patients treated with chemotherapy undergo premature menopause, and that the risk for premature menopause was found to be strongly associated with the age of the patient and the use of systemic chemotherapy [4]. The use of alkylating agents has been shown to increase the risk of premature ovarian failure nine-fold [5].

These findings have led the American Society of Clinical Oncology and the American Society for Reproductive Medicine to issue guidelines recommending that the potential effect of cancer treatment on fertility and options to preserve fertility be presented to the patient in the initial stages of treatment [6,7]. One of the suggested options for fertility preservation is oocyte cryopreservation. Although considered an experimental procedure, oocyte cryopreservation is available, with institutional review board (IRB) approval. It represents one of the few viable options for childbearing in reproductive-aged women undergoing cancer treatment, particularly in patients without a partner, or who have ethical or religious objections to embryo freezing [6].

Historically, oocyte cryopreservation has been hampered by poor oocyte survival. However, in recent years, the number of pregnancies resulting from fertilization of thawed/warmed oocytes has increased [[8-12], and [13]]. This success is due to a better understanding of oocyte physiology, the utilization of improved media and the implementation of new and innovative techniques [[14-17], and [18]].

Although the precise number of children resulting from cryopreserved oocytes is unknown, it is estimated that over 475 children have been born worldwide from oocytes that were frozen/vitrified and thawed/warmed, with no apparent increases in adverse postnatal outcomes such as low birth weight or an increased incidence of congenital anomalies [19,20].

However, despite these reassuring reports there are still concerns [[21-23], and [24]]. One of the contributing factors may be the lack of a comprehensive approach to evaluating the safety and efficacy of the use of cryopreserved oocytes. These concerns have been elucidated by recent guidelines and reports issued by ASRM's Committees report on this topic, which contend that, to date, the number of patients treated with oocyte cryopreservation, is inadequate to determine if the procedure has an effect on the development of children [25], and that because data is limited, the procedure should not be marketed as a method to defer reproductive aging [26]. Nonetheless, many women understandably have interest in this emerging technology and view oocyte cryopreservation as a strategy that may help them to realize their longer-term reproductive goals [27].

To date, there has been no systematic effort in the United States to collect outcomes of oocyte cryopreservation cycles on an ongoing basis. For this reason EMD Serono, Inc. (Rockland, MA, USA [an affiliate of Merck KGaA, Darmstadt, Germany]) is proposing the creation of a patient registry to track the safety and efficacy of oocyte cryopreservation procedures. Systematic collection of the data generated in cycles utilizing these procedures across a variety of clinics is critical to validate the technique and move the concept of oocyte cryopreservation from an experimental procedure to a proven and safe technique, thereby establishing this as a viable treatment option for fertility preservation.

The aim of Human Oocyte Preservation Experience (HOPE) Registry is to prospectively and systematically track the outcome of oocyte cryopreservation cycles to validate (1) the efficacy of techniques to freeze/vitrify and thaw/warm oocytes and (2) the safety of these procedures, in relation to the babies resulting from cryopreserved oocytes.

The HOPE Registry is a phase IV, prospective, multi-center, observational oocyte cryopreservation patient registry. Consistent with definitions and guidelines from the United States Agency for Healthcare Research and Quality, the HOPE Registry is a health services registry, an "organized system that uses observational study methods to collect uniform data to evaluate specified outcomes for a population defined by a particular disease, condition or exposure, and that serves one or more predetermined scientific, clinical, or policy purposes [28]." The data is purpose-driven and will be collected in a naturalistic manner. Patient management will be agreed upon by the caregiver and the patient together and, as such, there will be a defined ovarian stimulation protocol for each individual patient enrolled in the registry. The HOPE registry, like any other phase IV study, will follow a strict study protocol for patient enrollment and data collection, and will be listed on clinicaltrials.gov.

The goal of the three-year registry is to enroll approximately four hundred (400) women of reproductive age who will undergo thawing/warming of oocytes and subsequent embryo transfer. A pilot phase of The HOPE Registry opened in May 2008; formal enrollment is planned to begin in November 2008 (ASRM meeting, San Francisco, CA). All patients will be required to give written informed consent prior to enrollment in the registry. Local ethics committee or IRB approval will be required for each participating center, and the registry will be conducted according to the principles of good clinical practice and the Declaration of Helsinki.

Data will be collected, according to the protocol, via uniform, standardized forms for every enrolled patient and will continue for an additional 2 years to obtain birth outcomes from patients who achieved pregnancy during the third year. The HOPE Registry will be open to all investigators who would like to enroll, with the only requirement being the recording of data from the frozen/thawed oocytes that will be fertilized, and the culture and transfer of resulting embryos. During the course of the study prospective data will be entered in electronic case report forms, including demographics, controlled ovarian stimulation protocols, laboratory procedures to freeze and thaw oocytes, as well as embryo culture methods, embryo transfer procedures, implantation rates, biochemical and clinical pregnancy results, miscarriage and live birth rates. Child health and development after birth and at twelve (12) months of age will also be collected. Data related with genetic screening (preimplantation genetic diagnosis [PGD], amniocentesis, chorionic villus sampling, cord blood cells, etc.) will be utilized to monitor the normality of the genetic makeup of children born from oocyte cryopreservation cycles and to compare it with babies born from other established ART techniques.

The outcomes of all collected variables will be evaluated according to a pre-defined statistical analysis plan, and the cumulative results will be presented and discussed during an annual investigators' meeting. Following each meeting, the registry outcomes will be published so as to provide a widely accessible resource for patients and their caregivers regarding the efficacy of the different cyropreservation techniques, as well as the postnatal outcomes of the babies born from embryos generated from frozen/thawed oocytes.

## Discussion

The HOPE Registry will be the first prospective US registry to validate the efficacy and safety of oocyte cryopreservation techniques being utilized under IRB approval. Through the HOPE Registry, a real-world view of oocyte cryopreservation will be obtained, and with it, answers to many unresolved questions from healthcare providers and their patients who utilize this technique to preserve fertility for a variety of reasons. In the future, oocyte cryopreservation may become a tool for successfully preserving female gametes, facilitating the formation of oocyte cryobanks that transform the paradigm of oocyte donation, thereby bringing hope to women who would otherwise have no option for childbearing.

## Conclusion

The preservation of fertility has become an important issue, particularly in light of increasing survival rates in adolescent women and women of childbearing age with cancer, as the treatments can often impair fertility. Oocyte cryopreservation is one option for patients who have ethical or religious objections to embryo freezing. Unfortunately, the development of oocyte cryopreservation techniques has been hampered by a lack of a comprehensive approach to evaluate the safety and efficacy of these procedures. The present paper describes the establishment of the HOPE Registry, an endeavor to prospectively and systematically track the outcome of oocyte cryopreservation cycles.

## Competing interests

DE, FR, MC and JS are employees of EMD Serono Inc.

## Authors' contributions

DE made contributions to the conception and design, the drafts of the manuscript and gave final approval of the version to be published, FR, MC and JS made contributions to the conception and design and drafts of the manuscript.
